# Novel multiple tyrosine kinase inhibitor ponatinib inhibits bFGF-activated signaling in neuroblastoma cells and suppresses neuroblastoma growth *in vivo*

**DOI:** 10.18632/oncotarget.11580

**Published:** 2016-08-24

**Authors:** Haoyu Li, Yongfeng Wang, Zhenghu Chen, Jiaxiong Lu, Jessie Pan, Yang Yu, Yanling Zhao, Huiyuan Zhang, Ting Hu, Qing Liu, Jianhua Yang

**Affiliations:** ^1^ Department of Neurosurgery, Xiangya Hospital, Central South University, Changsha 410008, China; ^2^ The Institute of Skull Base Surgery and Neurooncology at Hunan Province, 410008, China; ^3^ Department of Microbiology, Peking University Health Science Center, Beijing 100191, China; ^4^ Texas Children's Cancer Center, Department of Pediatrics, Dan L. Duncan Cancer Center, Baylor College of Medicine, Houston, Texas 77030, USA; ^5^ Department of Ophthalmology, Shanghai Tenth People's Hospital, Tongji University School of Medicine, Shanghai 200072, P. R. China

**Keywords:** neuroblastoma, ponatinib, FGFR1, apoptosis, chemoresistance

## Abstract

Neuroblastoma (NB) is one of the most common pediatric malignancies in children. Abnormal activation of receptor tyrosine kinases contributes to the pathological development of NB. Therefore, targeting tyrosine kinase receptors to cure NB is a promising strategy. Here, we report that a multi-targeted tyrosine kinase inhibitor ponatinib inhibited NB cell proliferation and induced NB cell apoptosis in a dose-dependent manner. In addition, ponatinib suppressed the colony formation ability of NB cells. Mechanistically, ponatinib effectively inhibited the FGFR1-activated signaling pathway. Ponatinib also enhanced the cytotoxic effects of doxorubicin on NB cells. Furthermore, ponatinib demonstrated anti-tumor efficacy *in vivo* by inhibiting tumor growth in an orthotopic xenograft NB mouse model. In summary, our results showed that ponatinib inhibited NB growth both *in vitro* and *in vivo*.

## INTRODUCTION

Originating from the sympathetic nervous system, neuroblastoma (NB) is the most frequently diagnosed embryonic malignancy of childhood [1, 2] and it accounts for approximately 15% of all pediatric cancer-related deaths [3, 4]. Approximately half of NB patients have localized or locoregional tumors that are usually not aggressive and can be treated either with surgery alone or moderate doses of adjuvant chemotherapy. However, despite the progress in the biological understanding and treatment improvements made over the past decades, cure rate for patients with high-risk NB lag behind the overall survival rate of NB patients [5]. Therefore, developing novel chemo agents is of urgent need to cure this deadly disease.

Tyrosine kinases are a subgroup of the protein kinases family, which are responsible for the activation of many proteins by signal transduction cascades [6]. Phosphorylation of proteins by tyrosine kinases has been reported to play an important role in regulating cellular activities, such as cell survival, cell division, cell transformation etc. [7–9]. Mutations or dysfunction of tyrosine kinases can cause increased cell growth and migration, which frequently result in tumorigenesis and the progression of cancer [10–13]. Therefore, targeting oncogenic tyrosine kinase is a promising strategy in cancer therapy [14–16]. Tyrosine kinase inhibitors (TKIs) are a group of pharmaceutical drugs that selectively inhibits the activity of tyrosine kinases and thus are frequently developed as anticancer drugs [17–19].

Several studies have shown that the abnormal activation of receptor tyrosine kinases (RTKs) contributes to the development of various kinds of cancer types [20–22]. Aberrantly activated RTKs have been reported to promote NB tumor progression through activating signaling pathways such as PI3K/AKT/mTOR and JAK/STAT3 [23–26]. Furthermore, inhibition of some RTKs leads to increased apoptosis of NB cells [24]. However, current RTKs inhibitors have limited therapeutic outcomes [27, 28].

Ponatinib (trade name Iclusig, previously AP24534), a novel orally available tyrosine kinase inhibitor, has been developed using a computational and structure-based approach to treat chronic myelogenous leukemia (CML), acute myeloid leukemia (AML) and Philadelphia chromosome–positive (Ph+) acute lymphoblastic leukemia (ALL) [29–31]. Although BCR-ABL, an abnormal tyrosine kinase that is reported as the hallmark of CML and Ph+ ALL [32, 33], has been identified as the primary target for ponatinib, other targets including platelet-derived growth factor receptor α (PDGFRα), vascular endothelial growth factor receptor 2 (VEGFR2) and fibroblast growth factor receptor 1 (FGFR1) have also been identified to be inhibited by ponatinib [29]. As a multi-targeted tyrosine kinase inhibitor, ponatinib has been approved by the US Food and Drug Administration (FDA) for treating patients with resistant or intolerant CML and Ph+ ALL [34]. However, the anti-tumor efficacy of ponatinib has not been tested in NB yet.

Here in this study, to elucidate the cytotoxic effect of ponatinib on NB cells, we examined the efficiency of ponatinib *in vitro* against six NB cell lines and *in vivo* against the NGP xenograft mouse model. We found that ponatinib significantly inhibited NB cell proliferation and induced cell apoptosis in these NB cell lines by blocking FGFR1-activated PI3K/AKT/mTOR and JAK/STAT3 signal pathways. Ponatinib also suppressed the colony formation ability of a subset of NB cell lines. Moreover, ponatinib enhanced the cytotoxic effects of doxorubicin (Dox) on NB cells. Ponatinib also inhibited NB tumor growth and promoted cell apoptosis in an orthotopic xenograft NB mouse model. Together, our findings demonstrate that ponatinib inhibits NB growth both *in vitro* and *in vivo*, and that targeting multiple tyrosine kinases with small molecule inhibitors like ponatinib is a promising strategy for treating NB patients.

## RESULTS

### Ponatinib suppresses the cell viability in six NB cell lines

**Figure 1:**
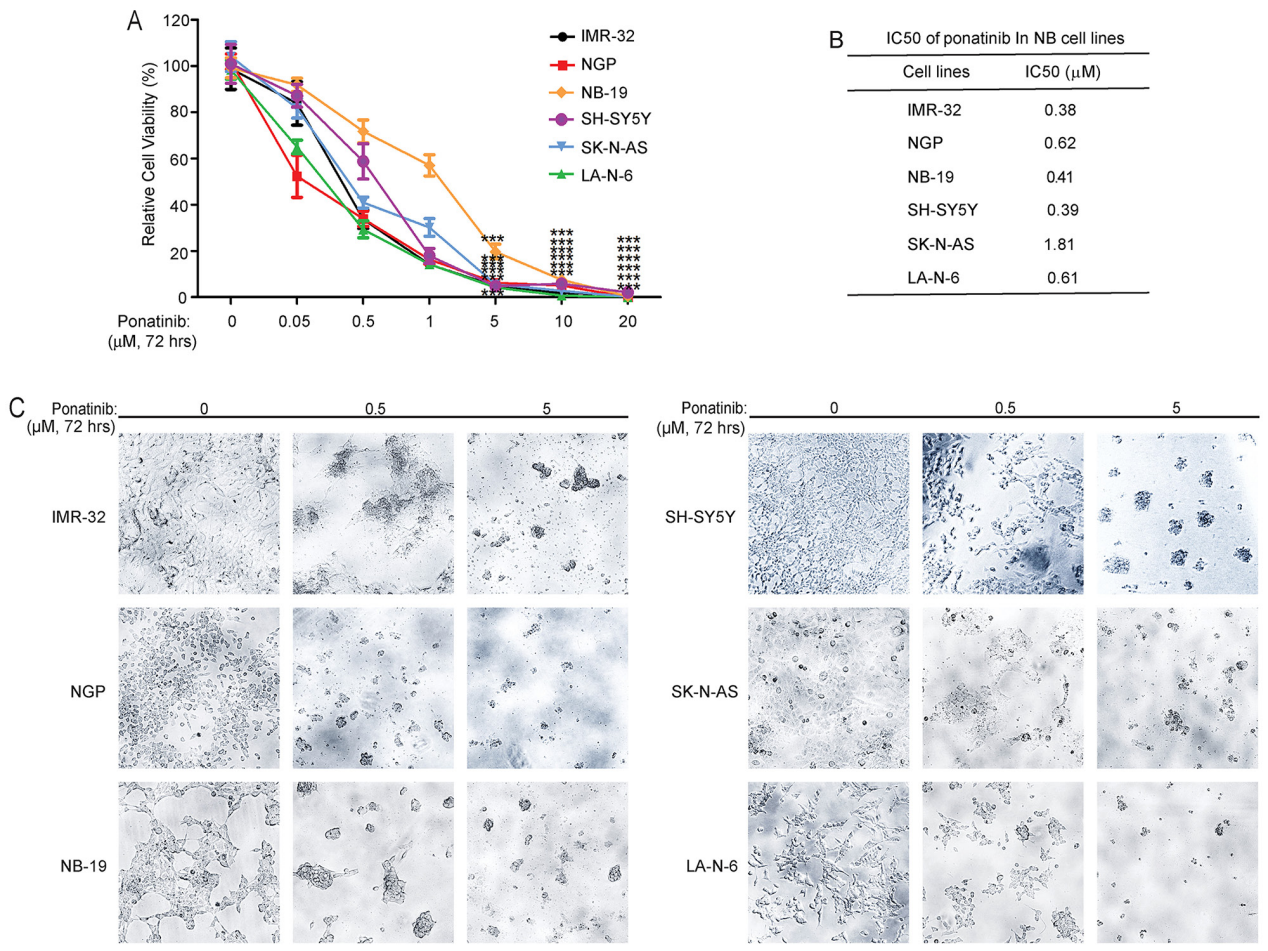
Ponatinib shows cytotoxic effects on NB cell lines **A**. Six NB cell lines, including the established chemoresistant NB cell line LA-N-6, were treated with increasing concentrations of ponatinib for 72 hrs. Cell viability was then assessed by adding a mixture of 10 μL of CCK-8 and 190 μL of RPMI and measuring absorbance at 450 nm. Data is represented as % vehicle ± S.D. *P*<0.001 (***) (Student's t-test, two-tailed) as indicated. **B**. The IC50 values of ponatinib in each cell line listed were calculated by using Prism 5.0, based on the data collected in the cell viability assay. **C**. Morphological changes of six different NB cell lines treated with increasing concentrations of ponatinib for 72 hrs were shown.

To explore the anti-tumor effect of ponatinib on NB cell lines, three MYCN-amplified cell lines (IMR-32, NGP and NB-19) and three MYCN-non-amplified cell lines (SH-SY5Y, SK-N-AS and LA-N-6) were exposed to increasing concentrations of ponatinib for 72 hrs. After that, the cell viabilities of the cells were detected by using the Cell Counting Kit-8. As shown in Figure 1A, ponatinib significantly inhibited the cell viability in all NB cell lines tested. Based on the cell viability data in Figure 1A, IC50 of ponatinib in different NB cell lines were calculated (Figure 1B). Cell morphology imaging after treatment also confirmed the cytotoxic effect of ponatinib on these NB cell lines (Figure 1C). Taken together, these data demonstrate that ponatinib can significantly suppress the viability of NB cell lines in a dose-dependent manner.

### Ponatinib suppresses colony formation potential of NB cells in soft agar

**Figure 2:**
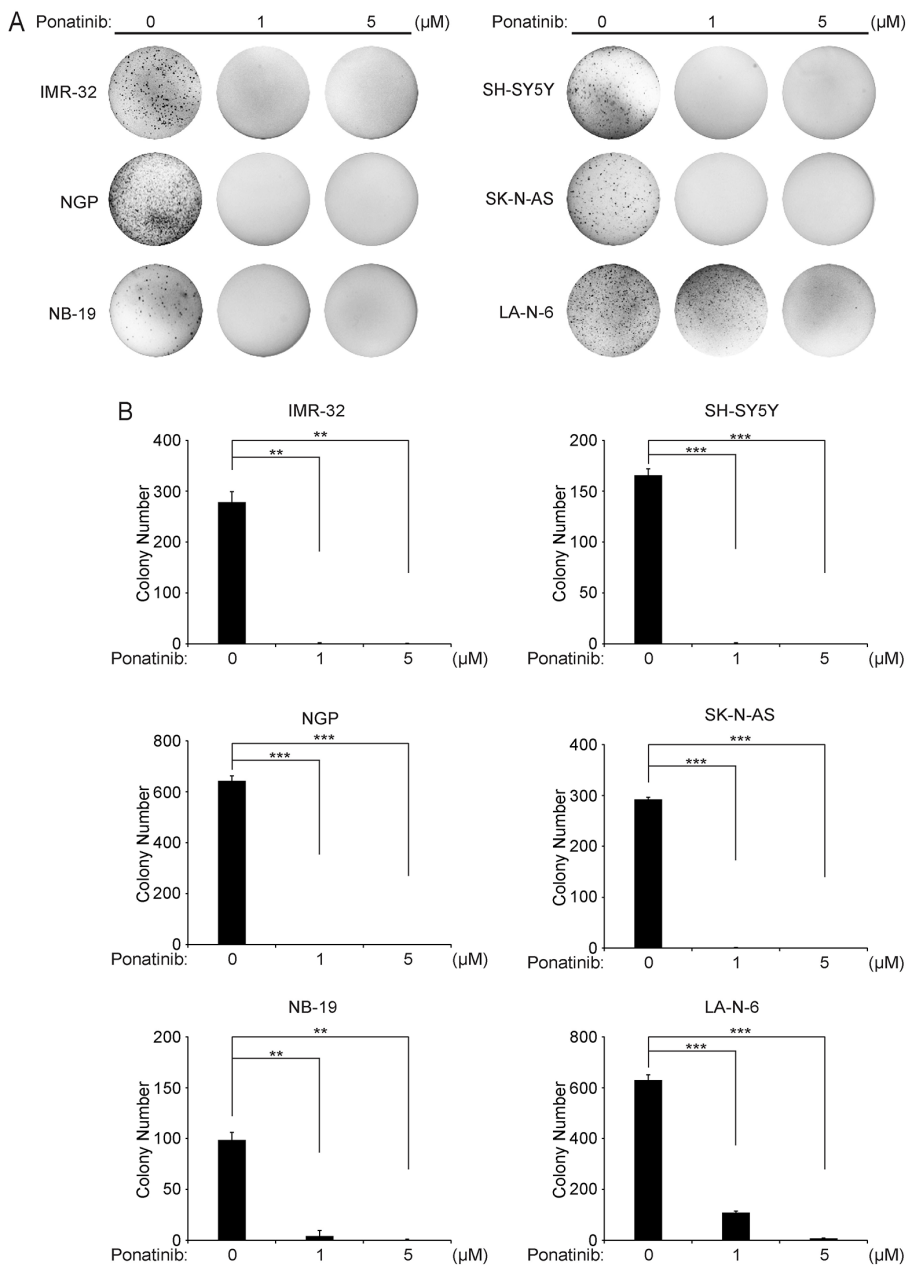
Ponatinib suppresses anchorage-independent growth of NB cells **A**. A panel of six NB cell lines were seeded in six-well plates with the indicated concentrations of ponatinib and agar, and grown for 2 to 3 weeks. Cells were stained with crystal violet for 4 hrs, and images were obtained. **B**. Colonies were counted and colony numbers were represented as % vehicle ± S.D. *P*<0.001 (***) (Student's t-test, two-tailed) as indicated.

Anchorage-independent growth in soft agar is one of the unique characteristics of tumor cells. Thus, to evaluate whether ponatinib could inhibit the colony-formation ability of NB cells, soft agar assays were performed using a panel of six NB cell lines. As shown in Figure 2A and Figure 2B, both the images and the colony counting results confirmed that ponatinib inhibited the anchorage-independent colony formation of all tested NB cells.

### Ponatinib effectively inhibits the activation of PI3K/AKT/mTOR and JAK/STAT3 signaling pathway by targeting FGFR1 in NB cells

**Figure 3:**
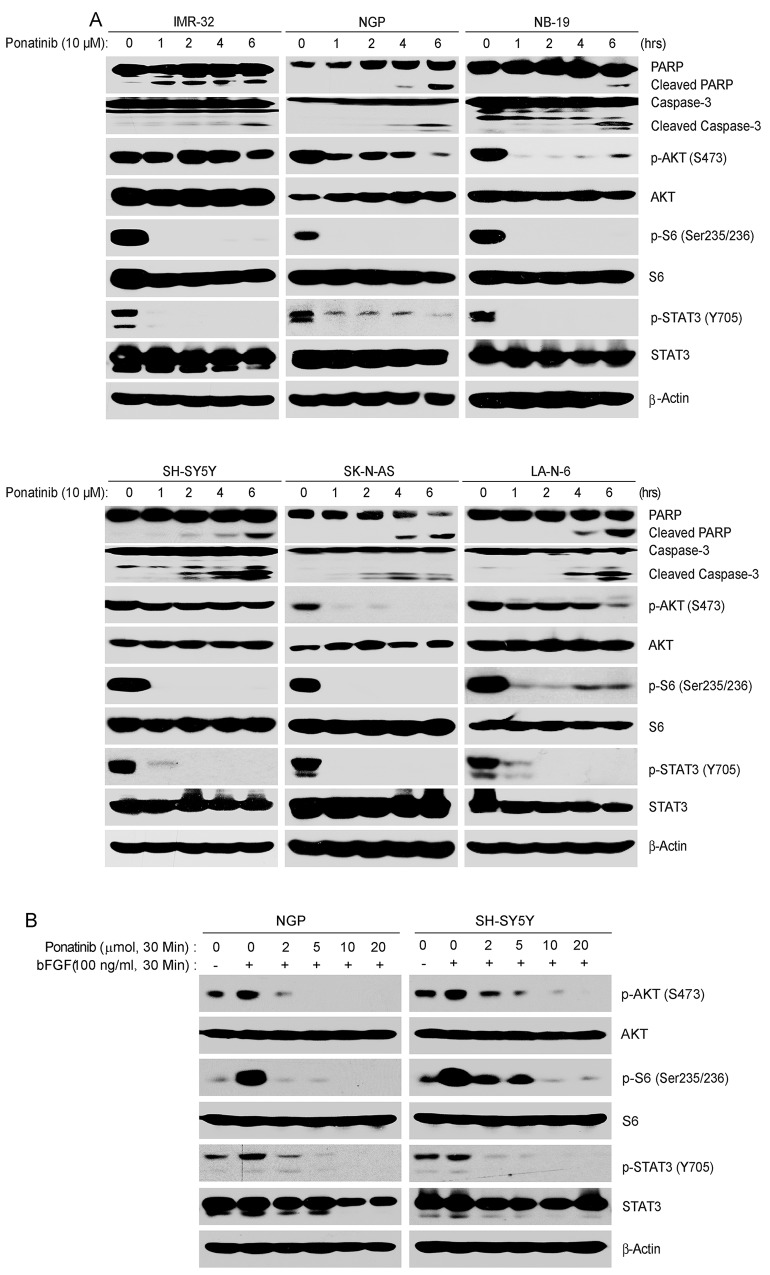
**A**. Ponatinib inhibits PI3K/AKT/mTOR and JAK/STAT3 signaling pathways, and induces cell death in NB cells. NGP, NB-19, SH-SY5Y, SK-N-AS and LA-N-6 cells were treated with 10 μM of ponatinib for various time points (0-6 hrs), subjected to SDS-PAGE, and then immunoblotted with PARP, p-AKT (S473), AKT, p-S6 (Ser235/236), S6, p-STAT3 (Y705), STAT3, Caspase-3, and β-Actin antibodies. **B**. Ponatinib inhibits the phosphorylation of the downstream effectors of FGFR1 after bFGF stimulation: NGP and SH-SY5Y cells were treated with different concentrations of ponatinib with or without 100 ng/ml bFGF after starvation for 16 hrs. Cells were harvested and subjected to SDS-PAGE, and then immunoblotted with the indicated antibodies.

PI3K/AKT/mTOR and JAK/STAT3 pathways play a vital role in tumor cell proliferation, and the inhibition of theses pathways contributes to apoptosis in tumor cells [35–37]. Since ponatinib targets multiple tyrosine kinases, we hypothesize that treatment with this drug could lead to effective inhibition of the PI3K/AKT/mTOR and JAK/STAT3 pathways. To investigate the mechanism that was responsible for the cytotoxic effects of ponatinib on NB cells, six NB cell lines (IMR-32, NGP, NB-19, SH-SY5Y, SK-N-AS and LA-N-6) were treated with ponatinib for various time points. As shown in Figure 3A, ponatinib significantly inhibited the phosphorylation of p-S6 (Thr235/236), and p-STAT3 (Y705) in a time-dependent manner in the six NB cell lines tested. However, in IMR-32 and SH-SY5Y cells, the inhibitory effect of ponatinib on the phosphorylation of AKT at Ser473 could only be observed after six hours of treatment. Ponatinib also induced cell apoptosis in NB cells by inducing PARP and Caspase-3 cleavages (Figure 3A).

FGFRs are the members of the RTK families and ponatinib potently inhibits FGFR1 activity [38]. To investigate whether ponatinib inhibits the FGFR1 signal pathway in NB cells, serum starved NGP and SH-SY5Y cells were treated with increasing concentrations of ponatinib for half an hour. And then the cells were exposed to basic fibroblast growth factor (bFGF) (100 ng/ml) in the presence and absence of ponatinib. The cells were harvested and the protein immunoblotting assay was performed with the indicated antibodies. As expected, the bFGF-induced phosphorylation of p-AKT (S473), p-S6 (Thr235/236) and p-STAT3 (Y705) were potently abolished by ponatinib in NB cells (Figure 3B).

### Ponatinib enhances the cytotoxic effect of Dox on NB cells

**Figure 4:**
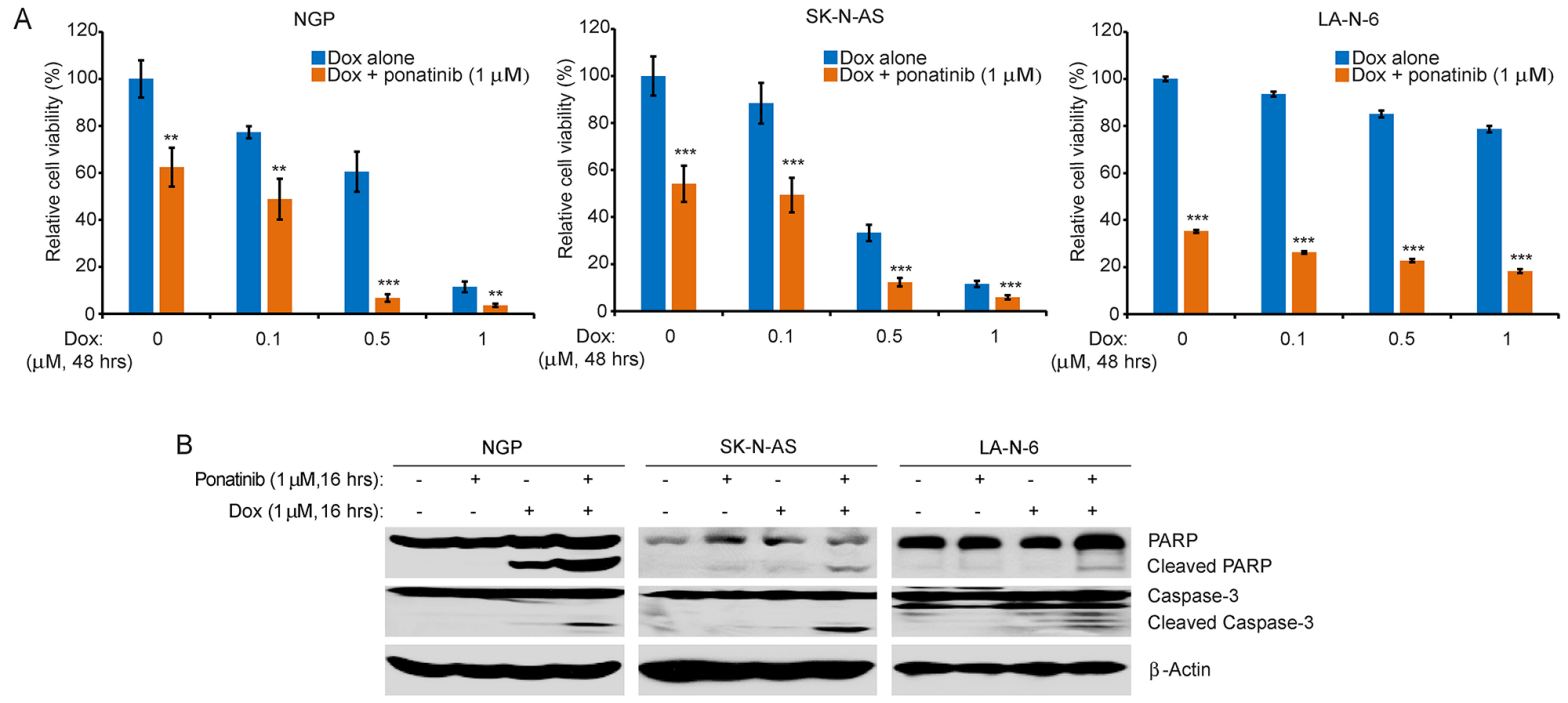
Ponatinib enhances the cytotoxic effect of Dox on NB cell lines **A**. NGP, SK-N-AS, and LA-N-6 cells were seeded in 96-well plates and were incubated with the indicated concentrations of Dox plus DMSO or 1 μM of ponatinib for 48 hrs. Cell viability was then measured by CCK-8 assay. **B**. NGP, SK-N-AS, and LA-N-6 cells were treated with either Dox (1 μM) alone, ponatinib (1 μM) alone, or their combination for 0-16 hrs, subjected to SDS-PAGE, and immunoblotted with PARP and Caspase-3 antibodies. β-Actin antibodies were used as a loading control for whole cell extracts in all samples.

Monotherapies are rarely effective in the treatment of high-risk NB due to chemoresistance. Thus, we evaluated the combination effect of ponatinib and Dox by using three NB cell lines, including the chemoresistant LA-N-6 cell line. Compared to Dox treatment alone, ponatinib significantly enhanced the cytotoxicity of Dox on all three cell lines tested (Figure 4A). Consistently, ponatinib enhanced Dox-induced cell apoptosis, as evidenced by increased levels of PARP and Caspase-3 cleavages (Figure 4B).

### Ponatinib shows anti-tumor efficacy in an orthotropic xenograft NB mouse model

**Figure 5:**
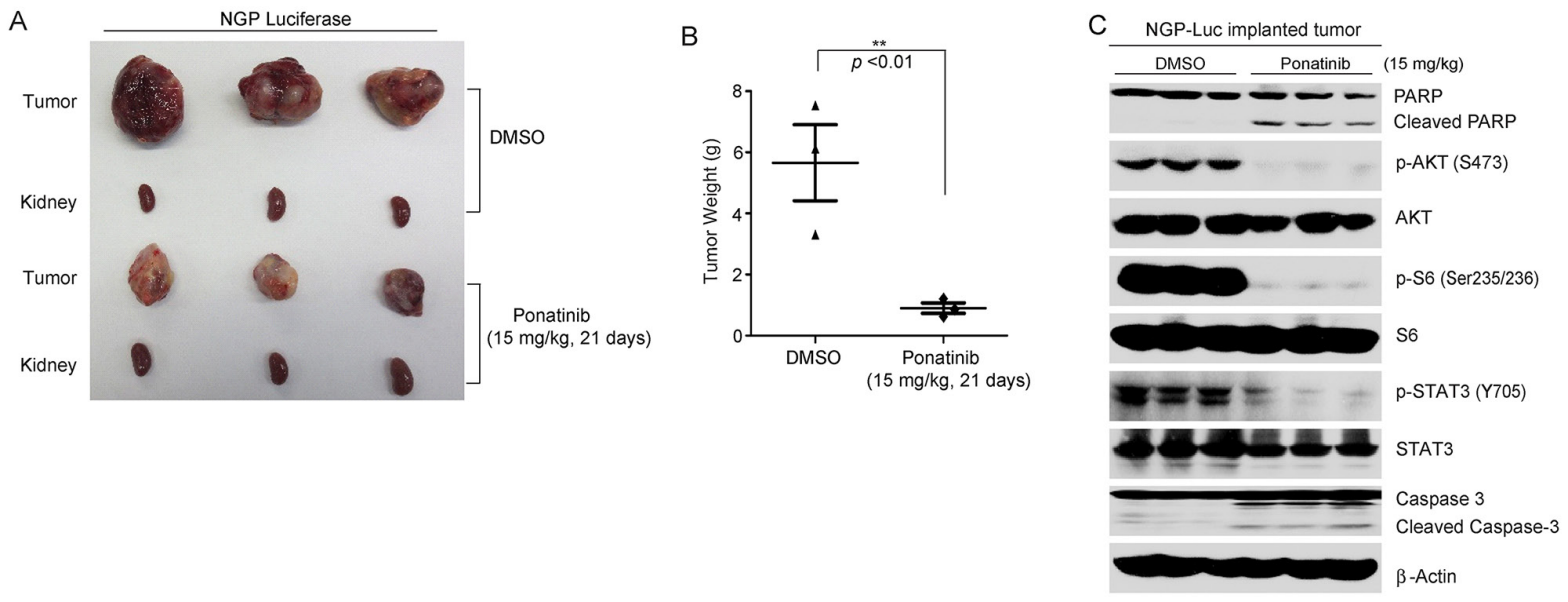
Ponatinib inhibits tumor growth in an orthotopic NB xenograft mouse model **A**. Photos of NGP xenograft tumors and the corresponding kidney controls from DMSO control group and ponatinib treatment group (15 mg/kg) were taken at the end of treatment (21 days). **B**. NGP derived tumor weights from control (N=3) and treatment groups (N=3) were presented. *P*<0.01 (**) (Student's t-test, two-tailed) was indicated. **C**. The mice bearing NGP xenografted tumors for four weeks were treated with DMSO or 15mg/kg of ponatinib by intraperitoneal injection once daily for two days, and then the tumors were harvested and subjected to SDS-PAGE and immunoblotted with indicated antibodies. β-Actin was detected as a loading control.

To test the efficacy of ponatinib *in vivo*, 1.0 × 10^6^ NGP cells with stable luciferase gene expression were separately implanted into the left kidneys of the nude mice. Two weeks after injection, tumor signals were captured and analyzed by bioluminescent imaging. Mice bearing similar size of tumors were randomly divided into two groups and then treated with either ponatinib (15 mg/kg) or an equal volume of dimethyl sulfoxide (DMSO) (carrier control). Ponatinib was administered by intraperitoneal (i.p.) injection daily for 21 days. At the end of the treatment, the xenografted tumors from control and treatment groups were dissected and weighed. Significant tumor growth inhibition was observed in the ponatinib treatment group compared to the control group (Figure 5A-5B).

In another set of xenograft mouse experiment, four weeks after NGP-luciferase cells implantation, the tumor bearing mice were treated with either ponatinib or DMSO via i.p. injection daily for two days. Then the tumor tissues were lysed and subjected to immunoblotting with the indicated antibodies. We found that ponatinib significantly blocked the phosphorylation of p-AKT (S473), p-S6 (Thr235/236), and p-STAT3 (Y705) in tumor tissues. Moreover, ponatinib caused cell apoptosis by inducing PARP and Caspase-3 cleavages in NB tumors (Figure 5C). These results suggest that ponatinib alone can effectively inhibit tumor growth by blocking the activation of PI3K/AKT/mTOR and JAK/STAT3 pathways in an orthotopic xenograft NB mouse model.

## DISCUSSION

The efficacy of ponatinib against multiple tyrosine kinases, such as ABL, PDGFRα, VEGFR2 and FGFR1 has been illustrated in several adult cancer types [28]. The FGFR signaling is a highly conserved signaling cascade and like other RTK family members, FGFR realizes its role by promoting several biologic processes which includes angiogenesis and regeneration [39]. Several studies showed that aberrant FGFR signaling is involved in the pathogenesis of cancer [40–42] and targeting FGFRs in cancer by small molecule inhibitors is a promising strategy in cancer therapy [43, 44]. FGFR1 is a member of the FGFR family. FGFR1 is reported to be prognostic biomarker in squamous cell carcinoma [45] and the abnormalities of FGFR1 has been shown to be associated with several cancer types including AML [46, 47]. To explore whether ponatinib-induced cytotoxicity results from the inhibition of FGFR1-activated signaling, we performed a bFGF stimulation assay and found that ponatinib inhibited bFGF-stimulated activation of FGFR1 signaling pathway. The results suggest that FGFR1-activated PI3K/AKT/mTOR and JAK/STAT3 signaling pathways may promote NB development and ponatinib-induced inhibition of FGFR1 signaling contributes to its anti-tumor effects.

Chemoresistance has been frequently raised as a critical issue in the clinical settings. In addition, the mutations of the RTKs family members anaplastic lymphoma kinase (ALK) and FGFR1 are reported to be associated with therapy-resistant relapses of the NB patients [48] and mutated ALK has been reported to contribute to the chemoresistance of NB [49, 50]. Since ponatinib could potently inhibit NB cell proliferation, which suggests that ponatinib may sensitize NB cells to Dox treatment. As shown in our study, ponatinib significantly enhanced Dox-induced cytotoxicity in NB cells. Therefore, it is possible that the combination therapy using ponatinib and Dox may overcome Dox-induced chemoresistance in NB therapy. Thus, in future studies, besides the use of single inhibitor, a combination therapy may be an option to benefit NB patients. In addition, using combination therapy with lower doses of ponatinib and Dox may achieve better treatment outcomes with less side effects.

Ponatinib was shown to be very effective in chemoresistant or intolerant CML patients in phase 1 and phase 2 studies, irrespective of mutational status [31, 51]. Unfortunately, the phase 3 clinical trial was terminated by the sponsor because of the accumulation of arterial occlusive events [52, 53]. The FDA temporarily suspended sales of ponatinib for “the risk of life-threatening blood clots and severe narrowing of blood vessels”, which may be the side effects beyond its anti-cancer effect. Moreover, it has been reported that the dose-intensity of ponatinib correlated with the incidence of adverse events [53]. Therefore, despite the significant anti-tumor effect caused by ponatinib in NB, the potential side effects of ponatinib should be taken into consideration before its application to NB patients. Since ponatinib targets several tyrosine kinases and only FGFR1-activated PI3K/AKT/mTOR and JAK/STAT3 signaling pathways have been investigated in this paper, it is highly likely that the inhibition of other targets also contributed to ponatinib-induced anti-tumor effect in NB. Thus, further study is needed to address such concerns.

In conclusion, ponatinib exhibits anti-tumor efficacy in NB by inhibiting FGFR1 signaling and blocking the activation of PI3K/AKT/mTOR and JAK/STAT3 signaling pathways, as evidenced by both *in vitro* and *in vivo* assays. Ponatinib also enhanced Dox-induced cytotoxicity in NB cells. Moreover, ponatinib showed anti-tumor efficacy in an orthotopic xenograft NB mouse model by blocking the activity of PI3K/AKT/mTOR and JAK/STAT3 signaling pathways. Collectively, our study provides compelling evidence that ponatinib is able to inhibit tumor growth as a single agent or combined with other therapeutic agents like Dox.

## MATERIALS AND METHODS

### Antibodies and reagents

Ponatinib was purchased from LC Laboratory (P-7022, Woburn, MA, USA). Purified mouse anti-basic FGF was purchased from BD biosciences (BDB610072, San Jose, CA, USA). Doxorubicin (Dox, D1515) and anti-β-Actin antibodies (A2228) were purchased from Sigma (Sigma-Aldrich Corp, St. Louis, MO, USA). The remaining antibodies—rabbit monoclonal p-AKT (Ser473) (4060S), rabbit polyclonal AKT (9272), rabbit monoclonal p-S6 (Ser235/236) ribosomal protein (4858S), rabbit monoclonal S6 ribosomal protein (2217S), rabbit monoclonal p-STAT3 (Y705) (9145L), rabbit monoclonal STAT3 (4904S), rabbit monoclonal PARP (9532S), rabbit polyclonal Caspase-3 (9662S), and anti-Mouse (7076S) or anti-Rabbit (7074S) IgG were purchased from Cell Signaling Technology (Danvers, MA, USA).

### Cell lines and cell culture

Five human NB cell lines: IMR-32, NGP, NB-19, SH-SY5Y, SK-N-AS were cultured in RPMI Medium 1640 (Lonza, Walkersville, MD, USA) supplemented with 10% (v/v) heat-inactivated Fetal Bovine Serum (FBS) (SAFC Biosciences, Lenexa, KS, USA), 100 units/mL penicillin, and 100 μg/mL streptomycin. The chemoresistant NB cell line LA-N-6 was grown in RPMI containing 20% (v/v) heat-inactivated FBS, 100 units/mL penicillin, and 100 μg/mL streptomycin. All cells were maintained at 37 °C in a humidified incubator with 5% CO_2_. All experiments were performed with cells under exponential growth conditions. The NGP cell line with a stable expression of luciferase was generated by transfection with a pcDNA3 luciferase expression plasmid into the cells. A stable cell line was established after 10 days of applying 800 μg/ml G418 selection (Enzo Life Sciences, Farmingdale, NY, USA).

### Cell viability assay

Cell viability assays were performed using the Cell Counting Kit-8 (CCK-8, WST-8 [2-(2-methoxy-4-nitrophenyl)-3-(4-nitrophenyl)-5-(2,4-disulfophenyl)-2 H-tetrazolium, monosodium salt]) (Dojindo Laboratories, Rockville, MA, USA). Cells were plated and grown in 96-well clear-bottom plates starting at 1 × 10^4^ cells/well. After 24 hrs of incubation, medium was changed and increasing concentrations of ponatinib, Dox, or their combinations were added to the wells. The cells were then incubated at 37 °C for 48 or 72 hrs. Then a mixture of 10 μL of CCK-8 and 190 μL of RPMI with 10% FBS was added into each well. After one hour of incubation, the absorbance was measured at 450 nm using a microplate reader. Each experiment was performed in replicates of six, and background reading of the medium was then subtracted from each well to standardize the results.

### Cell imaging

A total of six NB cell lines were separately seeded into 96-well plates at appropriate concentrations. After 72 hrs of treatment with indicated concentrations of ponatinib, cell morphologies were observed and captured using an optical microscope. Each result was performed in triplicate.

### bFGF stimulation

NGP and SH-SY5Y cells were plated and grown in RPMI-1640 medium supplemented with 10% FBS (v/v) for 24 hrs. The medium was then changed to FBS-free RPMI-1640 medium for 16 hrs. The serum starved NGP and SH-SY5Y cells were treated with increasing concentrations of ponatinib for 30 min before exposed to FBS-free RPMI-1640 medium with 100 ng/ml bFGF for half an hour. Cells were harvested and protein immunoblotting was performed.

### Colony formation assay

The soft agar assay was performed as described previously [54]. Briefly, a 5% (w/v) base agar layer was made by adding agar (214220, Difco Laboratories, Detroit, MI, USA) into distilled water. The mixture was autoclaved for 50 min and cooled in a 56 °C water bath. This solution was then mixed with RPMI with 10% FBS to a final concentration of 0.5%. To apply the bottom agar layer, 2 mL of the 0.5% agar/RPMI solution were added to each well and then cooled until semi-solid. For the top agar layer, each NB cell line was counted and added to 1.5 ml 0.3% agar at 1 × 10^4^ cells/well along with the indicated concentrations of ponatinib. Cells were grown at 37 °C for 2 to 3 weeks and then stained with 500 μL of 0.005% crystal violet (C3886, Sigma). Images were captured by microscope and colonies were counted after 4 hrs. Each assay was performed in triplicate.

### Protein immunoblotting

The protein immunoblotting assay (or western blot assay) was performed as previously described [55]. After each drug treatment, cells were washed twice with ice cold PBS and then lysed on a rotator in 4 °C for 30 min in a cooled RIPA buffer (50 mM Tris-HCl at pH 7.4, 150 mM NaCl, 1 mM EDTA, 1% NP-40, 0.25% sodium deoxycholate, 1 mM phenylmethylsulfonyl fluoride, 1 mM benzamidine, 10 μg/mL leupeptin, 1 mM dithiothreitol, 50 mM sodium fluoride, 0.1 mM sodium orthovanadate, and phosphatase inhibitor cocktail 2 and 3 (p5726 and p0044, Sigma)). After centrifuging at 13,000 rpm for 15 min, the supernatants were used as cell lysates. Protein concentrations were measured using Bradford reagent (Bio-Rad Laboratories, Hercules, CA, USA). Each sample was mixed 3:1 (v/v) with 4 × loading buffer and heated to 100 °C for 5 min. Lysates were then separated by SDS-PAGE, transferred to polyvinylidence fluoride (PVDF) membranes (Bio Rad), blocked with 5% milk or BSA for one hour at RT (25°C), and probed with appropriate dilutions of indicated primary antibodies overnight at 4 °C. The membranes were incubated with anti-mouse or anti-rabbit IgG conjugated with horseradish peroxidase at room temperature for 1hr. ECL-Plus Western detection system (GE Health Care, Buckinghamshire, UK) was then used for chemiluminescent visualization. β-Actin was used as a loading control for whole cell extracts.

### Orthotopic mouse model of NB

Five to six-week-old female athymic NCR nude mice were purchased from Taconic (Taconic, Hudson, NY, USA) and maintained under barrier conditions (pathogen-free conditions provided by plastic cages with sealed air filters). As described previously, the preclinical mouse model of NB was established using orthotopic (intrarenal) implantation of the NB cells [56, 57]. A transverse incision was created over the left flank of the nude mouse, and 1.0 × 10^6^ human luciferase-transduced NGP cells suspended in 0.1 ml of PBS were surgically injected into the left renal capsule and towards the superior pole of the left kidney of the nude mice. After allowing the cells to engraft for two to three weeks, mice bearing tumors of similar sizes (using bioluminescent imaging to monitor tumor growth) were randomized into treatment with DMSO or ponatinib (15 mg/kg/day) for 21 days. At the end of the treatment, all mice were dissected. Tumors and the right kidneys (control) were harvested and weighed. Another set of *in vivo* experiments were performed for the protein immunoblotting assay. Four weeks after implantation, NGP-luciferase cell xenografted mice were selected and treated with either DMSO or ponatinib via i.p. injection (15 mg/kg) once daily for two days. Tumors were then harvested for the protein immunoblotting assay. All mice were handled according to protocols approved by Institutional Animal Care and Use Committee of the Baylor College of Medicine.

### Statistical analysis

All values were presented as mean ± standard deviation (SD). A two-tailed Student's t-test was used to determine the statistical significance of *in vitro* and *in vivo* assays between the control and drug treatment groups. Each assay was repeated at least twice and representative results were presented. *P*<0.05 was considered to be statistically significant.
